# Disrupted Corticomuscular Coherence and Force Steadiness During Acute Low Back Pain

**DOI:** 10.3390/bioengineering12111269

**Published:** 2025-11-19

**Authors:** Franciele Parolini, Klaus Becker, Ulysses F. Ervilha, Rubim Santos, João Paulo Vilas-Boas, Márcio Fagundes Goethel

**Affiliations:** 1 Center for Rehabilitation Research (CIR), ESS, Polytechnic of Porto, Rua Dr. António Bernardino de Almeida, 4249-015 Porto, Portugal; rss@ess.ipp.pt; 2 Porto Biomechanics Laboratory, University of Porto, 4200-450 Porto, Portugal; klausmagnobecker@gmail.com (K.B.); ulyervil@usp.br (U.F.E.); jpvb@fade.up.pt (J.P.V.-B.); gbiomech@fade.up.pt (M.F.G.); 3 Center of Research, Education, Innovation and Intervention in Sport, Faculty of Sport, University of Porto, 4200-450 Porto, Portugal; 4 Laboratory of Physical Activity Sciences, School of Arts, Sciences and Humanities, University of São Paulo, São Paulo 05508-220, Brazil

**Keywords:** acute induced low back pain, encephalography, submaximal extensor force, electromyography

## Abstract

Background: Acute low back pain can impair motor control, yet its effects on force steadiness and cortical activity remain unclear. Methods: Thirty-three healthy adults (25 men, 8 women) performed a sustained spinal extension task at 20% of their maximum voluntary contraction under pre- and during-pain conditions induced by a hypertonic saline injection, as well as pre- and post-isotonic injection. Electromyography was recorded from the right and left longissimus muscles, and electroencephalography was collected from motor cortical areas. Spectral power in the alpha, beta, and gamma bands, along with corticomuscular and cortico-cortical coherence, was analyzed. Results: Acute pain reduced force steadiness and altered cortical activity, with increased beta and gamma band power in the prefrontal cortex and decreased alpha power in the motor cortex. Localized changes in corticomuscular coherence were observed in the Cz region (beta and gamma bands) during pain, suggesting nociceptive modulation of corticomuscular coupling. Conclusions: Experimentally induced acute low back pain disrupts motor control by reducing force steadiness and modifying cortical activation patterns, highlighting the interplay between pain and neuromuscular regulation.

## 1. Introduction

Low back pain is one of the most prevalent and disabling musculoskeletal conditions, significantly affecting motor control, functional and emotional steadiness [1,2,3]. Acute pain can alter neuromuscular coordination and strength steadiness, interfering with the interaction between the central nervous system and the muscles [4,5]. However, the impact of these changes depends on the type of task performed [2]. Voluntary tasks, which require precise cortical control, are more susceptible to these interferences, while postural tasks, which involve a greater contribution from subcortical and spinal circuits, seem to be less affected [6,7].

Force Steadiness is an essential marker of motor control, representing the ability to produce and maintain force consistently during sustained muscle contractions [8]. This ability is relevant in submaximal tasks that require postural control, such as spinal extension, where variability in force production can compromise functionality and motor efficiency [9,10]. In pain conditions, such as acute low back pain, steadiness can be affected by alterations in the neural mechanisms that regulate motor control [9,11]. The available results suggest that acute pain induces changes in the recruitment of motor units and in the coupling between cortical and muscular activity, which can increase force variability and impair motor performance [4,12,13].

In the neurophysiology of motor control, the mechanisms that support force maintenance under painful conditions remain unexplored. Although the literature has advanced in understanding the effects of pain on voluntary motor tasks, most studies focus on actions that do not fully capture the neural demands of everyday postural tasks [14,15,16,17,18]. Recent studies, such as the systematic review by [8], show that force stability plays a fundamental role in neuromuscular adaptation in response to pain. The studies suggest that postural tasks may employ distinct neural strategies, with less dependence on cortical activation, which may attenuate the effects of pain compared to tasks that require high voluntary motor control [15,19,20].

Corticomuscular coherence (CMC) reflects the degree of functional synchronization between cortical activity and muscle response, and is considered a robust marker of communication between the central and peripheral systems [20]. Changes in CMC may indicate neural adaptations due to pain, fatigue, or changes in motor control [15]. In parallel, corticocortical coherence (CCC) allows the evaluation of the integration between cortical areas involved in motor and cognitive pain regulation. Thus, the joint analysis of CMC and CCC provides a comprehensive insight into cortical modulation and neuromuscular coupling during motor tasks under nociceptive influence [14,20,21].

Corticomuscular coherence has been widely recognized as a robust and reliable approach to investigating the effects of acute low back pain on the neural mechanisms involved in motor control [15,20,22,23]. This coupling between cortical and muscular activity is modulated by several factors, including task demands, movement dynamics, and pain [16,18]. The relationship between acute pain and the ability to maintain steadiness in isometric tasks remains insufficiently explored [8]. This limits the understanding of the mechanisms by which the central nervous system adapts its motor behavior to preserve performance in the presence of pain [2,3,17].

To advance the understanding of pain-induced motor adaptations, this study adopts an innovative approach by integrating analyses of CMC and CCC. This framework enables the examination of central and peripheral neural adaptations during submaximal force steadiness tasks under experimentally induced acute low back pain. The primary endpoint was to assess the effects of acute pain on CMC and CCC as indicators of cortical modulation and functional connectivity. The secondary endpoint was to quantify changes in force steadiness, representing the behavioral manifestation of altered motor control. We hypothesized that acute pain would reduce force steadiness and CMC, indicating disrupted corticomuscular coupling, while increasing CCC, reflecting enhanced prefrontal–motor connectivity and compensatory cortical engagement during motor control under nociceptive conditions.

## 2. Materials and Methods

### 2.1. Study Design

This was a randomized crossover experimental study in which each participant attended two sessions, one involving experimental pain induction and another under control conditions, separated by a seven-day washout period to prevent carryover effects. The order of conditions was randomized across participants. The primary variables analyzed were CMC and CCC, reflecting cortical modulation and functional connectivity. The secondary variable was force steadiness, representing behavioral motor control.

The research protocol received ethical approval from the Ethics Committee of the Faculty of Sport, University of Porto (CEFADE 28-2023). All procedures were conducted in accordance with the Declaration of Helsinki. Prior to participation, all volunteers provided written informed consent after receiving detailed explanations about the study objectives and procedures.

### 2.2. Participant

Thirty-three individuals took part in this study. The inclusion criteria were defined as healthy individuals aged between 18 and 40, with no history of musculoskeletal diseases or recurrent pain in the past six weeks. Additionally, participants should not have used anti-inflammatory or analgesic medication in the 24 h prior to the experiment. Exclusion criteria included individuals with low back pain and pregnant women. These criteria were established to ensure that the participants represented a homogeneous population, thereby minimizing the impact of confounding variables on the study results. Among the subjects measured using a bioimpedance system (InBody 230, InBody Co., Ltd., Seoul, Republic of Korea), a total of 33 participants (aged 18–40 years; mean age 29.06 ± 5.96 years) were included in the study. Demographic characteristics of the participants are presented in Table 1.

### 2.3. Electromyography (EMG) Signals

EMG signals from the right and left longissimus muscles in the lower back, were collected using dEMG Galileo sensor (Delsys, Natick, MA, USA, serial number 017-03-01) electrodes, sizes 23 × 30 mm with four electrodes (5 mm of inter-electrode distance), 19 g of mass, and placed on dry skin. All the gears were plugged into an uninterruptible power supply with an external battery to isolate all the possible noise from the net wire. The active site of the sensor placement was cleaned and shaved, and the electrode was placed in the middle of the muscle belly, according to the SENIAM recommendations [24]. Further, the quality of the signal was checked based on two principles: the minimal offset amplitude accepted was around 20 and no more than 40 microvolts (µV) and the other caution was to check the signal-to-noise ratio, to ensure the quality of the signal collected. Galileo sensors were placed over the longissimus right and left muscles. Data collection was performed using the Delsys^®^ EMGWorks 4.8.0 software, common mode rejection ratio > 80 dB (Delsys, USA) with a sampling frequency of 2000 Hz.

### 2.4. Electroencephalography (EEG) Signals

All the recordings were acquired using Biopac Systems MP150 hardware with the following modules: MP150A.CE data acquisition unit, UIM100C universal interface module, and three EEG100C electroencephalogram amplifier modules. The software used for acquisition was AcqKnowledge 5.0. Participants wore a cap with a center on the vertex (point 0.0), determined by the International System 10/20 [24,25]. The fit was regularly monitored to ensure the accuracy of the positioning. The impedances of the sensors were monitored using Netstation software 5.5 (Electrical Geodesics, Inc., Eugene, OR, USA), considering a limit of 10 kΩ. Between tests, the impedances were checked and adjusted when necessary. EEG activity was amplified, sampled at 1000 Hz, and filtered with a bandpass filter (3–70 Hz). Subsequent analyses were carried out using MATLAB^®^ R2024a software.

For the coherence analyses, the electrodes Cz and Fp1 were selected based on their neuroanatomical relevance and functional associations with motor control and pain processing [20,26]. The Cz electrode, positioned at the vertex of the scalp, overlays the primary motor cortex, corresponding to the somatotopic representation of trunk musculature, particularly the lumbar paraspinal muscles involved in the isometric extension task. The Fp1 electrode, located over the left dorsolateral prefrontal cortex, was included in both cortico-cortical coherence (CCC) and CMC analyses. This region is implicated in the cognitive-affective modulation of pain, executive control, and attentional processes, all of which may influence descending motor pathways and alter sensorimotor integration under nociceptive conditions. Therefore, the inclusion of Fp1 enables the investigation of potential top-down regulatory influences on motor output and their modulation in the context of acute pain.

### 2.5. Experimental Setup and Protocol

#### 2.5.1. Force Test

##### Equipment Setup

The dynamometer was attached to a metal structure mounted on the treatment table to ensure stability. For trunk extensor strength testing, volunteers were positioned prone. The dynamometer was placed along the midline between the superior angles of the scapulae. Participants were instructed to perform spinal extension and maintain isometry for 30 s [27].

##### Familiarization

Five familiarization repetitions of the maximal voluntary contraction (MVC) test and three repetitions of the submaximal (20% of maximal) test with a trapezoidal signal were performed (steadiness) [8,28]. The intensity of 20% of the maximal voluntary contraction (MVC) was selected as a submaximal level requiring fine cortical control and shown to be particularly sensitive to detect changes in force steadiness and corticomuscular modulation under experimental pain [8,13,15,27].

##### Testing Procedure

**Figure 1:**
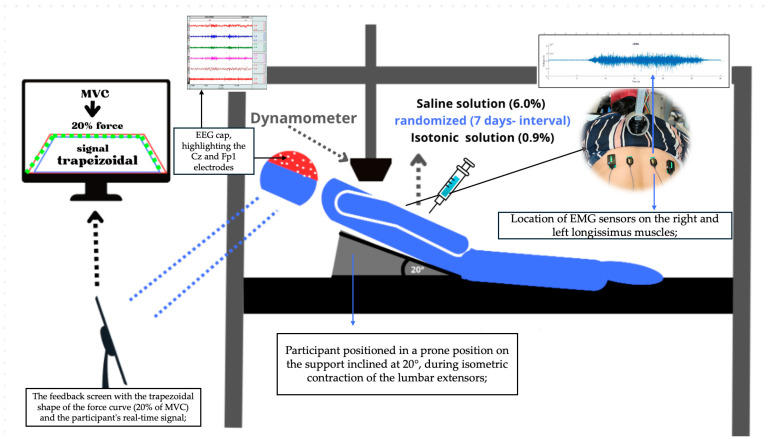
Experimental Setup: Screen display of the trapezoidal (20% of MVC), the trace is plotted as red line, and dashed green line is the real time signal performed by the volunteer using Delsys^®^ EMG Works 4.8.0 software, Volunteer performing trunk extension at 20°, (Delsys, Natick, MA, USA), dynamometer, EEG cap in Fp1 (pre-motor region) and Cz (motor-cortex area).

After a 2 min rest period, one MVC repetition was conducted, followed by 1 min of rest. Subsequently, a single repetition of the trapezoidal force signal was performed. Visual feedback of the force curve was provided throughout the trials. During the first visit, participants performed MVC followed by a submaximal isometric contraction at 20% MVC. The same procedure was repeated during a second visit, seven days later. The experimental setup is illustrated in Figure 1.

#### 2.5.2. Experimentally Induced Acute Low Back Pain

Experimental acute low back pain and a control condition were obtained, respectively, by intramuscular injections of hypertonic and isotonic saline solution, administered 7 days apart [29]. During the injections, the participants were positioned in the prone position on a wedge with a 20° incline, as illustrated in Figure 1. The inclination was measured in the cephalocaudal direction, while the spine was extended, which helped to promote muscle relaxation. The study employed a randomized crossover design, where each participant received a single 2.5 mL dose [30] of either hypertonic saline solution (6.0%) to induce pain, or isotonic saline solution (0.9%) [5], as a placebo, ensuring the test was conducted during the pain phase.

The injections were administered intramuscularly into the lumbar region at the level of the L3–L4 vertebrae, approximately 2–3 cm lateral to the midline. After cleaning the area with 70% alcohol, the solution was injected into the dominant side of the body using a 25 × 28 mm needle, at a depth of about 30 mm from the skin surface. Since hand dominance varied among participants, the dominant side did not correspond uniformly to the right or left anatomical side. Therefore, for consistency, subsequent analyses were organized according to the painful side (i.e., the side of injection) and the non-painful side (contralateral), regardless of individual anatomical laterality.

#### 2.5.3. Subjective Pain-Intensity Reporting

Pain intensity was assessed before initiation, continuously during the test, and at 1 min intervals up to a total duration of 7 min using the Numerical Pain Rating Scale (NPRS) [31], a widely validated and sensitive measure of subjective pain perception. The scale was anchored at 0 (no pain) and 10 (extremely severe pain). Participants reported their pain following standardized verbal instructions provided by the same researcher, ensuring consistent understanding and procedure. The NPRS ratings were self-reported and collected at fixed 1 min intervals throughout the 7 min period, which supported the temporal consistency and reliability of subjective pain reports [32].

### 2.6. Data Analysis

Preprocessing Force Signal

The force analysis was performed from the extracted and normalized data. Ten seconds of the plateau (stable phase) of the trapezoidal signal were analyzed during the sustained submaximal test. The period analyzed corresponded to the force stabilization phase, allowing an accurate assessment of sustained strength. Force steadiness was quantified as the coefficient of variation of the isometric force signal during the 10 s plateau phase, according to the equation:

(1) $$\mathrm{Force}\;\mathrm{Steadiness}=\frac{\mathrm{Standard}\;\mathrm{Deviation}\;\mathrm{of}\;\mathrm{Force}}{\mathrm{Mean}\;\mathrm{Force}}\times 100$$

This metric represents the relative variability of force and is expressed as a percentage (%CV). Lower CV values indicate higher steadiness and motor control, whereas higher values denote greater force variability. This approach follows previous recommendations for assessing motor steadiness in submaximal tasks [8].

Preprocessing Electromyography Signal

The force signal was digitally filtered using a fourth-order, zero-phase-lag Butterworth filter with a 10 Hz low-pass cutoff frequency. Electromyography signals were band-pass filtered between 20 and 400 Hz using a fourth-order Butterworth filter, followed by full-wave rectification. A low-pass filter at 50 Hz was then applied to the rectified signal to obtain the EMG envelope. Peak and integral EMG data were normalized to the maximum voluntary isometric contraction. Parameter extraction from individual trials followed a standardized sequence [33]:

- Variance (*VAR*, µV^2^) and Mean Absolute Deviation (*MAD*, µV) are statistical measures that assess data dispersion relative to the mean. Their calculations followed the following equation:

(2) $${VAR=\frac{1}{N}\sum_{i=1}^{N}\left(\begin{array}{cc} x_{i}\;- & \bar{x} \end{array}\right)}^{2}$$

b. *MAD* was calculated by the following equation:

(3) $$MAD=\frac{1}{N}\sum_{k=1}^{N}\left|x_{i}-\bar{x}\right|$$

c. EMG entropy was computed using the sample entropy (SampEn) algorithm, as defined by the following equation [34]:

(4) $$SampEn=-\log\left((\sum Ai\right)\left(\sum Bi)\right)=-\log\left(A∕B\right)$$

d. Full-wave rectification was applied to the EMG data, transforming all negative values into positive equivalents to generate a unidirectional representation of signal intensity.

(5) $$Int=\int_{a}^{b}f\left(x\right)dx\approx \left(b-a\right)\frac{f\left(a\right)+f\left(b\right)}{2}$$

e. The Integral of EMG (Int), quantifying the accumulated EMG signal, was computed using the trapezoidal rule, which approximates the area under the curve, providing a measure of the signal’s overall magnitude [4]:

(6) $$Int=\int_{a}^{b}f\left(x\right)dx\approx \left(b-a\right)\frac{f\left(a\right)+f\left(b\right)}{2}$$

f. Peak EMG was considered the maximum value of the filtered and rectified amplitude measured for the sample.

The selection of these six EMG outcome variables *VAR*, *MAD*, Entropy, Integral, peak amplitude, was based on their complementary ability to capture distinct dimensions of neuromuscular control. *VAR* and *MAD* reflect the dispersion and variability in motor unit recruitment, potentially altered by pain-related modulation of motor strategies. Entropy provides a nonlinear index of signal complexity and temporal regularity, offering insight into the adaptability of motor command. EMG integral captures the total neuromuscular output during contraction, while peak EMG reflects the highest level of muscle activation. Together, these features provide a comprehensive characterization of both the intensity and temporal structure of EMG activity, which are crucial for understanding the neuromuscular adaptations induced by acute experimental low back pain.

Coherence Analysis

Coherence analysis was performed using the discrete Fourier transform applied to successive segments of unprocessed EEG and EMG data, covering the entire frequency range. A Hanning window filter was applied to 1024 ms windows for spectral smoothing. Spectre Power (SP) was calculated using the following formula:

(7) $$SP\;c\left(f\right)=\;\frac{1}{n}\sum_{i=1}^{n}c_{i}(f)c_{i}^{*}\left(f\right)$$

Cortico-cortical coherence (CCC) was calculated between the CZ and Fp1 regions of the EEG signals recorded from the prefrontal and central areas of the motor cortex [14,35,36]. The analyses were performed on the two regions, Fp1 and Cz. The muscle-cortical coherence (CMC) was calculated between the unrectified EMG signals from the trunk extensor muscles in the lumbar region, specifically the right and left longissimus, and the EEG signals recorded from two electrodes located in the cortical motor area (Cz) and the premotor cortex (Fp1). The linear association between the cortical–cortical coherence (CCC) recordings between Fp1 and Cz, and between the EMG and EEG recordings (CMC) from the lumbar extensor muscles, at the selected cortical electrode sites, in the 12 to 45 Hz frequency range (including alpha, beta, and gamma bands), was assessed through coherence using the following equations [37]:

CMC or CCC₍c_1_,c_2_₎(f) = |S₍c_1_,c_2_₎(f)|^2^/(|SP₍c_1_₎(f)| |SP₍c_2_₎(f)|) (8)

where

(9) $${S\;}_{\left(C_{1},C_{2}\right)}\left(f\right)=\frac{1}{n}\;\sum_{i=1}^{n}c_{li}\left(f\right)c_{2i\left(f\right)}^{*}$$

Coherence was calculated between two signals, a first cortical signal (EEG) and a second cortical (CCC) or muscular (CMC) signal t each frequency f, where X(f) and Y(f) represent, respectively, the EEG and EMG (or EEG–EEG) signals, n is the number of segments analysed, and the symbol * indicates the complex conjugate. The value of Cxy(f) varies between 0 (no coherence) and 1 (maximum coherence).

Additionally, ii represents the segment number, with a total of nn segments, and the symbol * denotes the complex conjugate. This calculation returns a real value between 0 (no coherence) and 1 (maximum coherence). The confidence level was set at 95%. Coherence was considered significantly different from zero when the resulting value exceeded the desired confidence level (0.05) [38]. The CCC and CMC were quantified as the area under the coherence curve, separately for the alpha (8–12 Hz), beta (13–30 Hz), and gamma (30–45 Hz) frequency bands. This calculation was performed for each electrode combination (Cz and Fp1) and for each of the two trunk extensor muscles. The EEG power for both electrode locations was quantified as the area under the spectral power curve, also separately for the same frequency bands.

The synchronization between the EEG and EMG signals was performed by means of a digital trigger automatically sent by the EMGworks^®^ software (Delsys, Inc.) to the EEG system. This trigger allowed for precise temporal alignment of the signals, which is essential for spectral coherence analyses, which require rigorous simultaneity between time series.

### 2.7. Statistical Analysis

A descriptive analysis of electromyography (EMG) variables was performed, including measurements of variance, MAD, integral, entropy, and peak for the right and left longissimus muscles under both isotonic and hypertonic conditions. Additionally, the alpha, beta, and gamma bands of EEG activity were analyzed at the Fp1 and CZ positions under the same experimental conditions. Initially, the Shapiro–Wilk normality test was conducted to assess the data distribution.

Given that normality was violated, the Wilcoxon signed-rank test was applied to all variables under isotonic and hypertonic conditions, comparing the pre- and post-condition measurements for each muscle group and each EEG band. Due to the number of comparisons performed, a Bonferroni correction was applied to control for Type I error. Effect sizes were calculated using the coefficient r derived from the Wilcoxon *Z* value (r = Z/√N) [39]. The magnitude of effect sizes was interpreted as small (r = 0.10), medium (r = 0.30), or large (r = 0.50) [40]. All analyses were performed using SPSS Statistics software (IBM Corporation, Armonk, NY, USA, Version 27). Data are presented as median and interquartile range (IQR) in both text and figures.

## 3. Results

**Figure 2:**
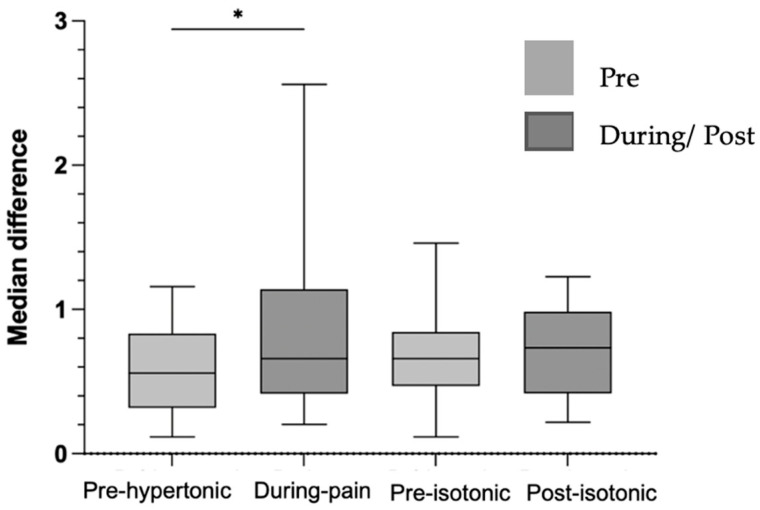
Graph of the median difference in force steadiness in %, pre-hypertonic, during pain, pre and post-isotonic. Legend: * < 0.05.

Force steadiness showed a median of 0.65% (IQR 0.51) in the pre-hypertonic condition and 0.55% (IQR 0.72) during acute induced low back pain in the submaximal task. Force steadiness decreased significantly during pain (*Z* = –2.55, *p* = 0.011, r = 0.44), indicating a medium effect size and suggesting a reduction in force steadiness control during low back pain (Figure 2). In contrast, in the isotonic protocol, force steadiness showed no significant differences, in force steadiness control following the isotonic protocol.

### 3.1. Electromyography

**Figure 3:**
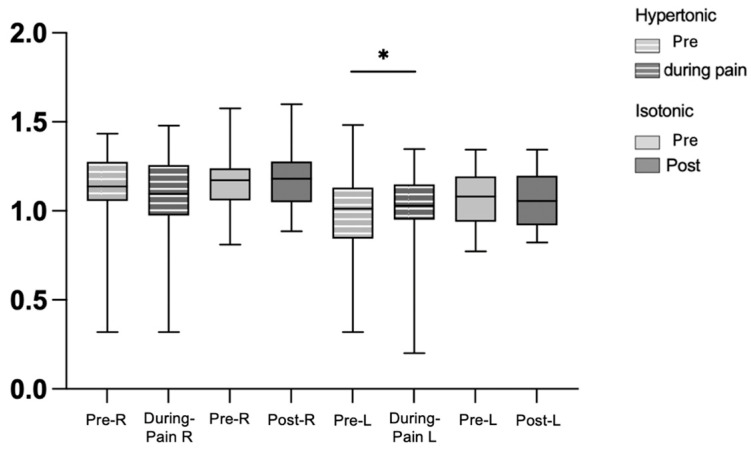
Graph of entropy in force steadiness, pre-hypertonic, during pain, pre- and post-isotonic, in the right and left longissimus muscles. Legend: * < 0.05. right longissimus muscle (R), left longissimus muscle (L).

In the electromyography analysis, the variance, MAD, integral and peak of the right and left longissimus muscles did not show statistically significant differences between the pre- and post-isotonic condition. Similarly, these variables did not differ between the pre-hypertonic conditions and during acute induced low back pain for both the right and left longissimus muscles. However, only entropy showed a statistically significant difference in the hypertonic condition during pain in the left longissimus (Figure 3), pre-hypertonic (1.09 (IQR 0.28–1.09)) and during low back pain (1.13 (IQR 0.21–1.09) with *p* = 0.032), suggesting changes in the complexity of the EMG signal associated with pain.

### 3.2. Cortical–Cortical Coherence

In the hypertonic condition before and during pain, as well as in the pre- and post-isotonic condition, there were no significant changes in cortical coherence between Fp1 and CZ in the three frequency bands analyzed on both sides (right and left longissimus) with *p* > 0.05, with no substantial changes in cortical activity in these conditions.

### 3.3. Corticomuscular Coherence

**Figure 4:**
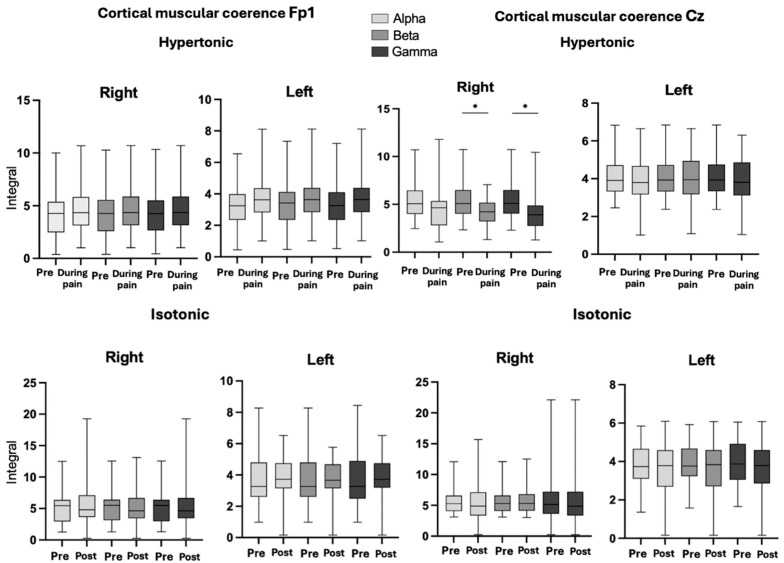
Graph of CMC, in conditions hypertonic (pre- and during pain), pre- and post-isotonic, in the right and left longissimus muscles, in Fp1 and Cz regions in band Alpha, Beta, and Gamma. Legend: * < 0.05. Band Alpha, Beta and Gamma.

The *p*-values for the CMC analysis in the isotonic and hypertonic conditions in the right and left longissimus muscles were analyzed in different frequency bands (alpha, beta and gamma) in two specific regions: Fp1 and CZ, for both the right and left of the longissimus muscle (Figure 4). On both sides, right and left in Fp1, no significant differences were observed between the hypertonic conditions, in the alpha (*p* = 0.40), beta (*p* = 0.45) and gamma (*p* = 0. 53), as well as in the pre- and post-isotonic conditions (alpha, *p* = 0.86; beta, *p* = 0.69; gamma, *p* = 0.79 on the right side and alpha, *p* = 0.86; beta, *p* = 0.85; gamma, *p* = 0.83 on the left side), maintaining CMC during the task. At Cz, there were significant differences only in the right longissimus muscle in the beta (*p* = 0.018) and gamma (*p* = 0.007) bands, during the induced pain.

### 3.4. Eeg Power Spectra

The results indicate variations in alpha, beta, and gamma frequency bands in the Fp1 and Cz regions across the different experimental conditions, as presented in Table 2. In the Fp1 region, alpha band power significantly increased during pain compared to the pre-hypertonic condition, followed by a reduction in the pre- and post-isotonic conditions. Beta band power also showed a substantial increase during pain, followed by a sharp decrease in the post-isotonic condition. Gamma band power exhibited a decreasing trend across conditions, reaching its lowest values in the post-isotonic condition.

In the Cz region, alpha band power progressively decreased across conditions, reaching its lowest value in the post-isotonic condition. Beta band power showed a marked reduction during pain, followed by a slight recovery in the post-isotonic condition. Gamma band power remained relatively stable during pain but decreased significantly in the post-isotonic condition. These findings indicate changes in cortical activity across the different experimental phases.

Pain intensity, measured using the NPRS, was significantly higher during the pain condition compared to the no-pain condition (median = 4.71; IQR = 1.61 vs. 0.00; Z = 4.92, *p* < 0.001, r = 0.90). Similarly, the placebo condition showed elevated scores relative to the no-pain condition (median = 1.50; IQR = 1.00 vs. 0.00; Z = 4.12, *p* < 0.001, r = 0.75). Twenty-four hours later, all participants reported complete pain resolution (NPRS = 0).

## 4. Discussion

This study offers a new perspective on the neurophysiological adaptations underlying motor control during experimentally induced acute low back pain, with a focus on force steadiness. Pain significantly compromised motor accuracy, increasing force production variability and suggesting an adverse impact on the neural regulation of fine motor skills [8]. The significant reduction in CMC in Cz during pain indicates nociceptive modulation of the functional connectivity between the motor cortex and the muscles involved in the task [15,20,41,42].

At the same time, EEG spectral analysis revealed cortical reorganization patterns, with increased alpha and beta band activity in Fp1, suggesting compensatory recruitment of cortical networks associated with sensorimotor integration and fine motor control. Concurrently, the suppression of alpha band power in Cz reinforces a specific neural adaptation to nociceptive processing. These findings highlight a highly dynamic interaction between motor regulation and nociceptive processing, challenging classical models of voluntary motor control and emphasizing the need for approaches that integrate the influence of nociception on movement biomechanics.

### 4.1. EMG Entropy

The changes in the entropy of the electromyographic signal indicate neurophysiological adaptations in motor control, especially in the left longissimus muscle (contralateral to the painful side) during pain, suggesting a process of cortical compensation in motor control on this side. This increase in entropy means an increase in signal complexity, due to greater heterogeneity of patterns. These changes may reflect a functional reorganization, in which the brain seeks to improve neurofunctional efficiency and postural stability in response to the painful stress experienced on the affected side [13,43]. These results are consistent with the idea that pain activates compensatory mechanisms, which include both cortical recruitment of motor areas and areas associated with executive control [14,44]. This can be interpreted as an attempt by the neuromuscular system to compensate for pain or to respond to changes in the motor demands of the submaximal task [45]. These changes are associated with the nervous system’s adaptation to pain, with implications for motor control and force steadiness, possibly leading to increased inefficiency in muscle control during task execution [43].

### 4.2. Modulation of CMC in Steadiness During Low Back Pain

Analyzing the CMC associated with the alpha, beta, and gamma frequency bands reveals how the cortical system adapts to a demanding task, maintaining submaximal contraction in the force steadiness. The reduction in CMC in the beta and gamma band, in the motor cortex, indicates a dysfunction in the transmission of information between the motor cortex and the muscles, possibly due to the increased cognitive load involved in the task affected by pain [14,15,46]. The fact that the right longissimus muscle (where pain was induced) showed a decrease in CMC, particularly in the beta and gamma bands, while the left side showed an increase in entropy, reveals a lateralized cortical response to pain. This decrease in CMC reflects a challenge in motor coordination, which suggests that pain not only affects motor control but also overloads the cognitive processes needed to sustain the motor task [13,16,17].

### 4.3. Comparison of CCC Pain and Placebo

According to Poortvliet et al. (2019) [15], CCC is particularly sensitive to variations in the type of motor control required by a given task. In our study, the submaximal task may not have been sufficient to robustly activate the cortical networks responsible for coherence at specific frequencies. Studies suggest that during submaximal efforts, cognitive and neuromuscular demands do not reach the intensity necessary to elicit a significant increase in CCC, particularly in the alpha, beta, and gamma bands [14,20,41,47]. From a similar perspective [46] indicate that under painful conditions, the brain prioritizes motor protection mechanisms by redistributing control to subcortical networks or altering cortical activation patterns, thereby limiting detectable changes in CCC [22,42]. The overall lack of statistical significance across the investigated conditions may reflect the complex interaction between task type, the nature of experimentally induced pain, and the adaptive dynamics of cortical motor control, as suggested by the current literature [14,20,41].

### 4.4. Low Back Pain Adaptations in EEG

One of the interesting findings is the differentiated modulation of cortical activity in the premotor (Fp1) and motor (Cz) regions, highlighting the multifaceted neural strategies employed to maintain force stability under painful conditions. Increased alpha and beta activity in Fp1 suggests greater involvement of the prefrontal cortex in sensory-motor integration, possibly reflecting the cognitive demand required to regulate movement in the face of nociceptive interference [2,6,47,48]. At the same time, the suppression of alpha and beta oscillations at Cz, accompanied by increased gamma activity, indicates intracortical inhibition and increased motor excitability, respectively, of the adaptive mechanism that probably facilitates force maintenance in the presence of pain [6,47]. These findings corroborate recent models suggesting that the motor cortex undergoes task-specific reorganization when faced with nociceptive stimuli, to guarantee functional stability even in adverse conditions [15,49].

Essentially, our findings challenge traditional conceptions about the impact of pain on motor performance, highlighting its influence on neuromuscular modulation and adaptation. Rather than causing a ‘simple drop’ in neuromuscular function, acute low back pain appears to trigger a remodeling of motor control, characterized by targeted cortical adaptations and a strategic redistribution of neuromuscular resources [50]. This is evidenced by the modulation of CMC, where changes in connectivity between the motor cortex and the lumbar muscles suggest a refined compensatory mechanism that enables force stability despite sensory disturbance [51]. This pattern of cortical modulation that we found aligns with one of the neurophysiological biomarkers described by [49] as part of the cortical signature predictive of individual pain sensitivity, which reinforces the importance of alpha oscillation dynamics in regulating motor behavior in pain contexts [2,48].

The contralateral response observed, with differential adaptations between muscle groups (right and left longissimus), highlights the specificity and complexity of these regulatory processes [15,47,49]. These neurophysiological adaptations go beyond the immediate motor response and have significant implications for clinical practice. The nervous system’s ability to dynamically reconfigure its control strategies in response to acute pain suggests that rehabilitation approaches must overcome simplistic models of motor inhibition. Instead, interventions should take advantage of this adaptive potential, promoting neuromuscular strategies that optimize strength, stability and posture. Furthermore, by identifying individualized cortical signatures of pain-related motor adaptation, as highlighted in recent literature [49,52], it becomes possible to improve therapeutic protocols by tailoring interventions to target specific neural mechanisms involved in movement efficiency in painful conditions.

Lastly, this study elevates our understanding of the relationship between pain, strength stability, and neural control, in how we conceive motor adaptation in acute musculoskeletal pain. The evidence presented not only fills a critical gap in the literature but also paves the way for innovative therapeutic strategies that exploit neuroplasticity to mitigate the functional impact of low back pain. The treatment of acute low back pain, based on neurophysiological adaptations, can promote recovery and postural stability of the lumbar muscles and, when combined with biopsychosocial factors [1], can decrease the risk of recurrence of low back pain [3,53]. These findings represent a significant advance in the field, reinforcing the urgency of integrating strength stability assessments into both research and clinical practice, unlocking new opportunities for optimizing motor function in individuals facing acute pain.

### 4.5. Limitations

Although this study has made important contributions to understanding the neurophysiological adaptations in motor control during experimentally induced acute low back pain, as reflected in force steadiness, some limitations should be acknowledged. The sample consisted of only healthy individuals, which may limit the results for populations with chronic low back pain or other clinical conditions. Additionally, the lack of a longitudinal assessment prevents the analysis of the persistence or adaptation of cortical patterns over time, limiting the understanding of pain dynamics and the evolution of cortical adaptation mechanisms.

### 4.6. Future Research

Future studies should consider including more diverse populations, such as individuals with chronic low back pain, to assess whether the patterns observed in healthy participants extend to clinical conditions. Longitudinal designs would be valuable to investigate how cortical and motor adaptations evolve over time with repeated pain episodes. Additionally, combining experimental models with clinical assessments may provide a more comprehensive understanding of pain mechanisms, bridging the gap between controlled acute pain studies and the complex pathophysiology of chronic low back pain.

## 5. Conclusions

Acute low back pain impaired force steadiness, reflecting altered motor control strategies. Neuromuscular adaptations, including increased complexity of muscle activation and lateralized changes in corticomuscular coherence, indicate compensatory mechanisms to maintain muscle force steadiness under painful input. Region-specific cortical reorganization, evidenced by modulation of brain activity in motor and premotor areas, highlights dynamic interactions between sensorimotor and cognitive networks, supporting the preservation of motor performance during pain.

## Figures and Tables

**Table 1:** Demographic characteristics of study participants.

Variable	Women (n = 8)	Men (n = 25)
Age (years)	29.77 ± 6.20	28.79 ± 5.90
Height (cm)	164.66 ± 6.30	175.20 ± 5.50
Body mass (kg)	65.14 ± 6.20	80.00 ± 9.30

**Table 2:** The table shows the inhibition or excitation of the bands in the and Cz regions, in the alpha, beta, and gamma bands, in the pre-hypertonic, during pain, and pre- and post-isotonic conditions. Legend: median (IQR—interquartile range), ** *p* < 0.01, * *p* < 0.05, in both conditions: hypertonic before and during pain, and pre- and post-isotonic.

VARIABLE	Pre-Hypertonic	During of Pain	Pre-Isotonic	Post-Isotonic
	Fp1			
ALPHA *	0.04 × 10^−3^ (IQR-0.09 × 10^−3^)	5.87 (IQR-1.0)	1.71 (IQR-0.08)	1.87 (IQR-0.11 × 10^−6^)
BETA *	0.07 × 10^−3^ (IQR-0.21 × 10^−4^)	8.23 (IQR-1.0)	1.35 (IQR-0.02)	7.86 × 10^−9^ (IQR-1.10 × 10^−10^)
GAMMA *	7.88 × 10^−6^ (IQR-1.00 × 10^−6^)	2.12 × 10^−8^ (IQR-0.1 × 10^−7^)	1.82 × 10^−9^ (IQR-9.4 × 10^−11^)	6.23 × 10^−12^ (IQR-1.23 × 10^−13^)
	Cz			
ALPHA *	6.78 × 10^−7^ (IQR-1.00 × 10^−7^)	3.21 × 10^−8^ (IQR-0.1 × 10^−8^)	7.42 × 10^−9^ (IQR-4.49 × 10^−10^)	2.31 × 10^−10^ (IQR-1.23 × 10^−11^)
BETA *	1.23 × 10^−6^ (IQR-4.8 × 10^−10^)	2.98 × 10^−11^ (IQR-6.66 × 10^−12^)	1.26 × 10^−11^ (IQR-6.03 × 10^−13^)	9.85 × 10^−10^ (IQR-1.22 × 10^−11^)
GAMMA **	3.01 × 10^−9^ (IQR-1.01 × 10^−12^)	3.34 × 10^−9^ (IQR-4.69 × 10^−10^)	3.80 × 10^−11^ (IQR-1.47 × 10^−11^)	2.04 × 10^−12^ (IQR-9 × 10^−15^)

## Data Availability

The data presented in this study are available on request from the corresponding author. The data are not publicly available due to privacy.
